# Elucidation of the biosynthetic pathway of *cis*-jasmone in *Lasiodiplodia theobromae*

**DOI:** 10.1038/s41598-017-05851-7

**Published:** 2017-07-27

**Authors:** Ryo Matsui, Naruki Amano, Kosaku Takahashi, Yodai Taguchi, Wataru Saburi, Hideharu Mori, Norio Kondo, Kazuhiko Matsuda, Hideyuki Matsuura

**Affiliations:** 1 Research Faculty of Agriculture, Hokkaido University, Sapporo, 060-8589 Japan; 2 Graduate School of Agriculture, Faculty of Agriculture, Kinki University, Nakamachi, Nara 631-8505 Japan

## Abstract

In plants, *cis*-jasmone (CJ) is synthesized from α-linolenic acid (LA) via two biosynthetic pathways using jasmonic acid (JA) and *iso*-12-oxo-phytodienoic acid (*iso*-OPDA) as key intermediates. However, there have been no reports documenting CJ production by microorganisms. In the present study, the production of fungal-derived CJ by *Lasiodiplodia theobromae* was observed for the first time, although this production was not observed for *Botrytis cinerea*, *Verticillium longisporum*, *Fusarium oxysporum*, *Gibberella fujikuroi*, and *Cochliobolus heterostrophus*. To investigate the biosynthetic pathway of CJ in *L*. *theobromae*, administration experiments using [18,18,18-^2^H_3_, 17,17-^2^H_2_]LA (LA-d5), [18,18,18-^2^H_3_, 17,17-^2^H_2_]12-oxo-phytodienoic acid (*cis*-OPDA-d5), [5′,5′,5′-^2^H_3_, 4′,4′-^2^H_2_, 3′-^2^H_1_]OPC 8:0 (OPC8-d6), [5′,5′,5′-^2^H_3_, 4′,4′-^2^H_2_, 3′-^2^H_1_]OPC 6:0 (OPC6-d6), [5′,5′,5′-^2^H_3_, 4′,4′-^2^H_2_, 3′-^2^H_1_]OPC 4:0 (OPC4-d6), and [11,11-^2^H_2_, 10,10-^2^H_2_, 8,8-^2^H_2_, 2,2-^2^H_2_]methyl *iso*-12-oxo-phytodienoate (*iso*-MeOPDA-d8) were carried out, revealing that the fungus produced CJ through a single biosynthetic pathway via *iso*-OPDA. Interestingly, it was suggested that the previously predicted decarboxylation step of 3,7-didehydroJA to afford CJ might not be involved in CJ biosynthesis in *L*. *theobromae*.

## Introduction

**Figure 1:**
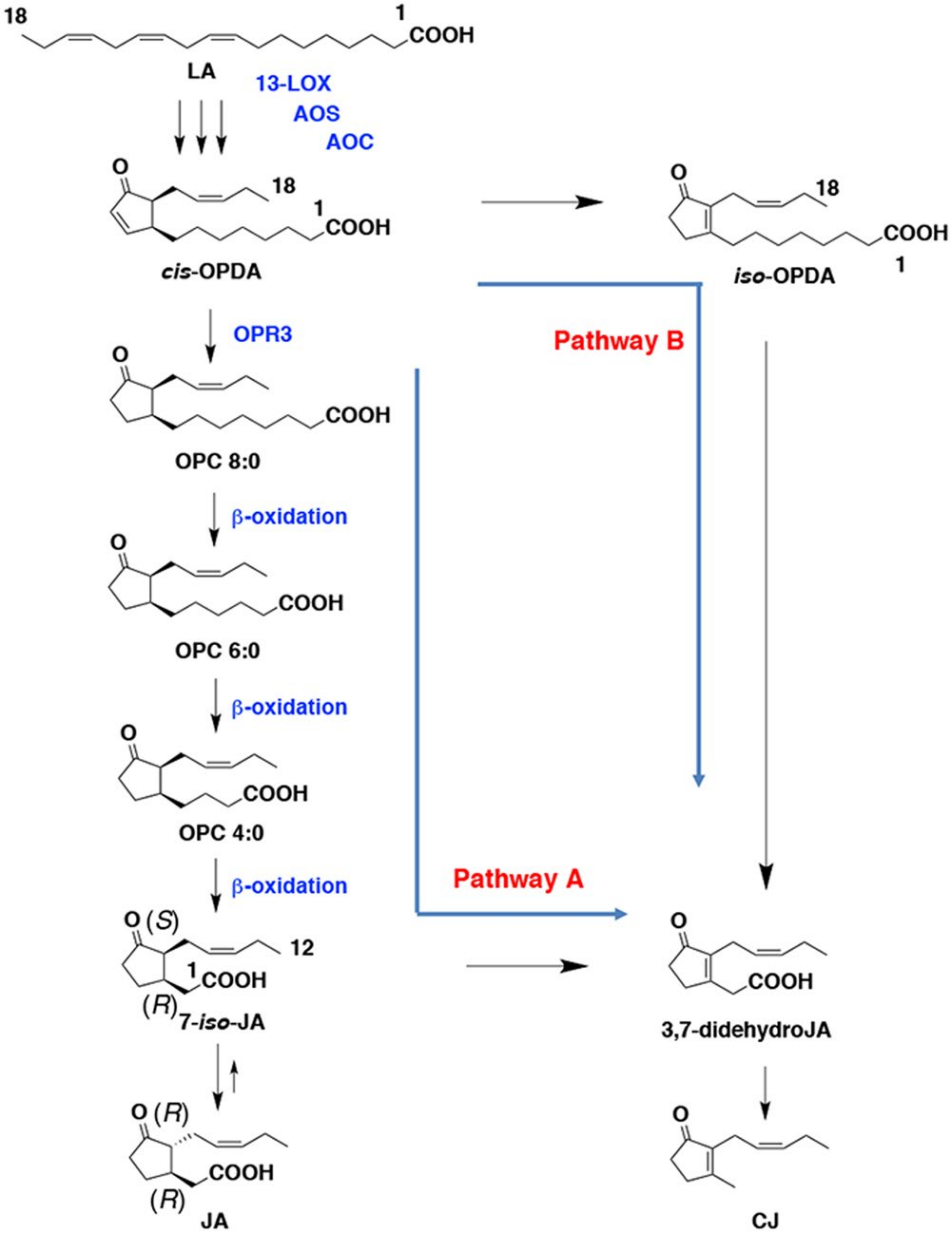
Biosynthetic pathway to give CJ and JA. LA: α-linolenic acid, OPDA: 12-oxo-phytodienoic acid, OPC 8:0: 3-oxo-2-(2′-[*Z*]-pentenyl)-cyclopentane-1-octanoic acid, OPC 6:0: 3-oxo-2-(2′-[*Z*]-pentenyl)-cyclopentane-1-hexanoic acid, OPC 4:0: 3-oxo-2-(2′-[*Z*]-pentenyl)-cyclopentane-1-butanoic acid (OPC 4:0). 13-LOX: 13-lipoxygenase, AOS: alene oxide synthase, AOC: alene oxide cyclase, OPR3: 12-oxophytodienoate reductase 3.

Jasmonic acid (JA) is a phytohormone and key mediator in plant wound responses to insects, necrotrophic pathogens^1–3^ and other environmental stresses. The biosynthesis of JA begins with the oxygenation of α-linolenic acid (LA) in the chloroplast to give (+)-7-*iso*-JA in the peroxisome^4^ via 12-oxo-phytodienoic acid (*cis*-OPDA), 3-oxo-2-(2′-[*Z*]-pentenyl)-cyclopentane-1-octanoic acid (OPC 8:0), 3-oxo-2-(2′-[*Z*]-pentenyl)-cyclopentane-1-hexanoic acid (OPC 6:0), and 3-oxo-2-(2′-[*Z*]-pentenyl)-cyclopentane-1-butanoic acid (OPC 4:0). (+)-7-*iso*-JA is readily epimerized to afford JA, which has an absolute configuration of (3*R*, 7*R*) (Fig. 1). The synthesized (+)-7-*iso*-JA is metabolized to afford other jasmonates, including MeJA, 12-hydroxy-JA (12-OH-JA), and JA-amino acid conjugates. It has been generally accepted that the compounds derived from oxygenated polyunsaturated fatty acids are categorized as oxylipins, and thus, JA and its derivatives are members of this family. JA-related compounds are well known, and their biological functions have been reported^5, 6^. Among of them, jasmonoyl isoleucine (JA-Ile) is an important player due to its curtail biological roles to interact with its receptor, COI1^7–10^, which needs to induce JA dependent biological phenomena. However, Yan *et al*.^11^ reported that JA-Ile and other four JA conjugates are the ligand of the JA receptor. Methyl jasmonate (MeJA) is a volatile compound involved in the response to wound stress, and volatized MeJA is thought to induce defensive proteins in plants^12^.

*cis*-Jasmone (CJ) is one of oxylipins, and it was reported that the biosynthetic pathway of CJ in plant was proceeded using JA as a biosynthetic intermediate (pathway A, Fig. 1)^13^. In this report, [7-^2^H_1_, 5,5-^2^H_2_, 2,2-^2^H_2_]JA and [9-^2^H_1_, 8-^2^H_1_]methyl 3,7-didehydrojasmonate were converted into deuterium-labeled CJs in jasmine flowers and the leaves of six higher plants, including lima beans (*Phaseolus lunatus*) and willow (*Salix alba*). It was proposed that the conversion of 3,7-didehydroJA to CJ is achieved by decarboxylation^13^. Moreover, a recent study has shown that *iso*-12-oxo-phytodienoic acid (*iso*-OPDA) is an early precursor of CJ^14^, suggesting biosynthesis via pathway B (Fig. 1). *iso*-OPDA is an isomer of *cis*-OPDA, an important intermediate in the JA biosynthetic pathway (Fig. 1). Interestingly, the successful conversion of labelled *iso*-OPDA into CJ by the yeast *Saccharomyces cerevisiae* has also been reported, although the yeast had no operative pathway for CJ. Several reports have demonstrated the biological activities of *cis*-OPDA^15, 16^. The existence of OPDA-Ile in *Arabidopsis thaliana* was proven^17^, the related biological activity was evaluated by Arnold *et al*.^18^. However, no similar reports exist for *iso*-OPDA. There is an interesting report about *iso*-OPDA as a metabolite, which revealed that *cis*-OPDA isomerase is involved in phytohormone detoxification in the insect gut, a process by which *cis*-OPDA is converted to *iso*-OPDA^19–23^. It has been generally accepted that CJ is involved in plant defense systems, similar to JA. For example, CJ is released from wounded leaves, attracts aphid parasitoids and acts as a repellent of the pest^24, 25^. CJ is also released from the flowers of many plants, such as jasmine, neroli (*Citrus bigaradia*), jonquil (*Narcissus jonquilla* L.), bergamot (*Citrus bergamia*), and the *Pittosporum* family. On the other hand, some insects such as butterflies^26^ and silk worms^27^ use CJ as a sex pheromone. Therefore, potential agricultural use of CJ in applications such as aphid control has been studied^28^.

Plant pathogens produce various secondary metabolites, and some fungi produce phytohormones, such as JA, auxin, and abscisic acid^29^. It was initially thought that fungi produce phytohormones to disturb signaling systems of plants and enable facile invasion of plants. The fungus, *Verticillium longisporum* uses CORONATINE INSENSITIVE1 (COI1), which is an essential protein for establishing the JA-dependent wound response, to invade plants, and thus, it was hypothesized that the fungus might synthesize JA-related compounds. *Fusarium oxysporum* causes vascular wilt disease in more than 100 plant species and reportedly produces 22 kinds of JA analogues^30^. Furthermore, Thatcher and coworkers discovered that *F*. *oxysporum* also hijacks COI1-mediated jasmonate signaling to promote disease development in *Arabidopsis* ^31^. The fungus *Botrytis cinerea*, *Gibberella fujikuroi* and *Lasiodiplodia theobromae* also synthesize JA, and the JA biosynthetic pathway in *L*. *theobromae* has been reported by Tsukada *et al*.^32^. *Cochliobolus heterostrophus* can cause corn leaf blight in maize and produces a death acid that is an analogue of JA^33^. The biosynthetic pathway of this death acid is similar to that of JA, and the fungus reportedly uses the plant’s JA biosynthetic pathway to synthesize the death acid. However, fungal production of CJ has not been reported. In this study, we discovered that *L*. *theobromae* produces CJ. Furthermore, we used a combined approach involving feeding the fungus deuterium-labeled compounds and gas chromatography–mass spectrometry (GC-MS) analysis to elucidate the CJ biosynthetic pathway in *L*. *theobromae*.

## Results and Discussion

### Screening of fungal producers of CJ

**Figure 2:**
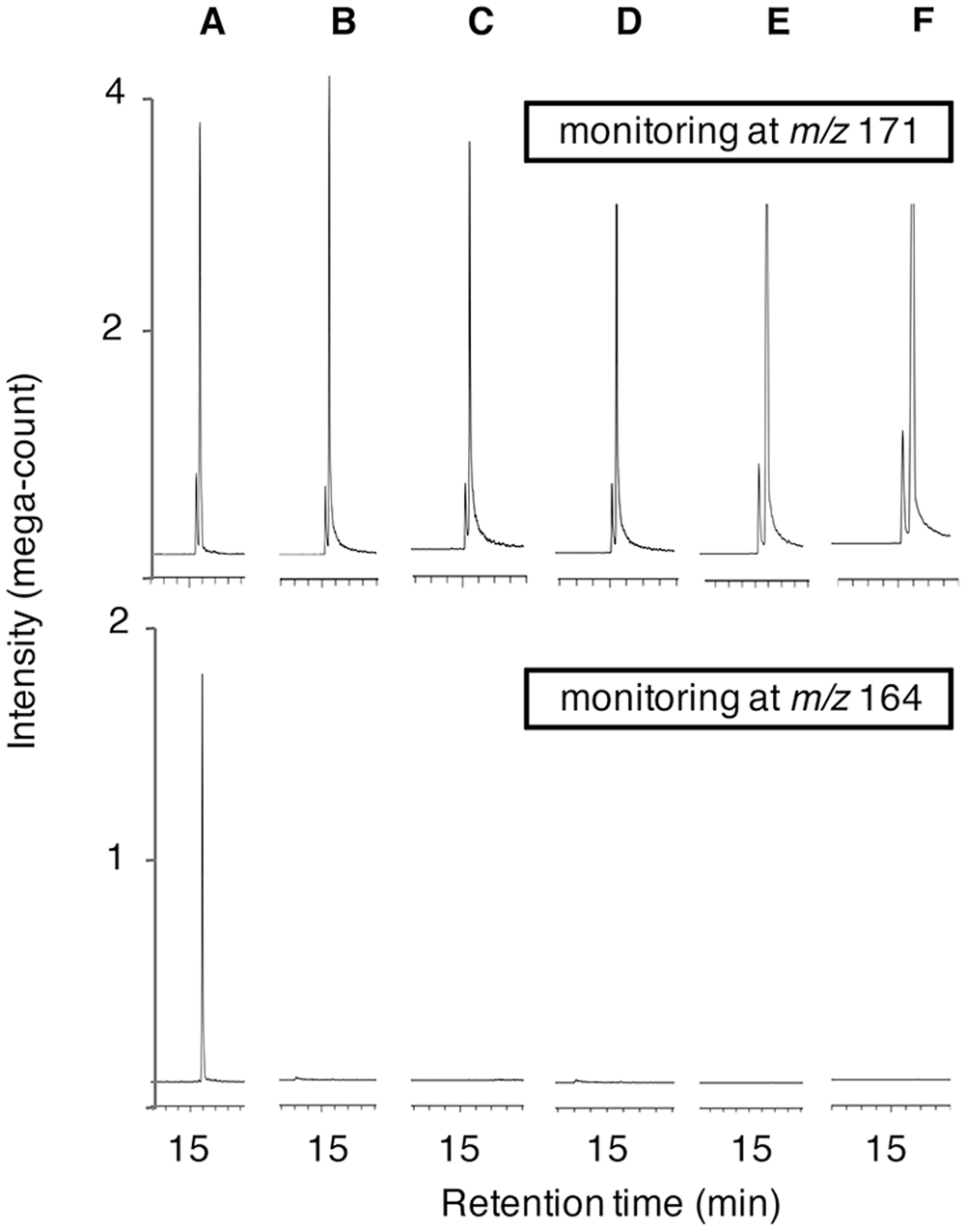
Representative GC MS chromatograms analyzing fungal derived *cis*-jasmone. (**A**,** B**,** C**,** D**,** E** and **F**) measuring fungal derived *cis*-jasmone using selected ion monitoring at *m*/*z* 164 (lower) and 171 (upper, internal standard) for *L*. *theobromae*, *B*. *cinerea*, *V*. *longisporum*, *F*. *oxysporum*, *G*. *fujikuroi*, and *C*. *heterostrophus*, respectively.

The similarities in the chemical structures of CJ and JA suggest that some species of plant pathogens that synthesize JA or its related compounds could produce CJ. Thus, the ability of six species of plant pathogens, *L*. *theobromae*, *B*. *cinerea*, *V*. *longisporum*, *F*. *oxysporum*, *G*. *fujikuroi*, and *C*. *heterostrophus*, to synthesize CJ was evaluated using GC-MS. To ensure accuracy in the experiment, [4,4-^2^H_2_, 3,3-^2^H_2_, 1,1,1-^2^H_3_]CJ (CJ-d7, *m*/*z* 171) was synthesized according to a reported method^34^, except that commercially available CJ was used as the starting material. The isotopic purity was established by comparing the data of GC-MS for CJ and CJ-d7. The incorporation of ^2^H_2_ was found to be 94% for CJ-d7. Representative GC-MS/MS chromatograms for authentic CJ are shown in Supplementary Fig. 1, and the results of screening experiment are given in Fig. 2. Peaks corresponding to CJ-d7 (*m*/*z* 171), an internal standard, in selected ion monitoring mode were detected in the extracts of all the six species of fungi (Fig. 2A–F, upper panels). However, a peak corresponding to CJ (*m*/*z* 164) was only detected in the culture filtrate derived from *L*. *theobromae* (Fig. 2A, lower panel), and the corresponding MS pattern agreed with that of the authentic standard shown in Supplementary Fig. S1. However, the peak corresponding to CJ was not detected in the filtrates derived from the other five pathogens (Fig. 2B–F, lower panels). A concentration of CJ, calculated based on the peak area ratio between CJ-d7 and fungal-derived CJ, was determined to be 8.7 μg/mL in the culture filtrate of *L*. *theobromae*.

### Metabolism of deuterium-labeled LA-d5 and *cis*-OPDA-d5 to MeJA-d5 and CJ-d5 in feeding experiments

**Figure 3:**
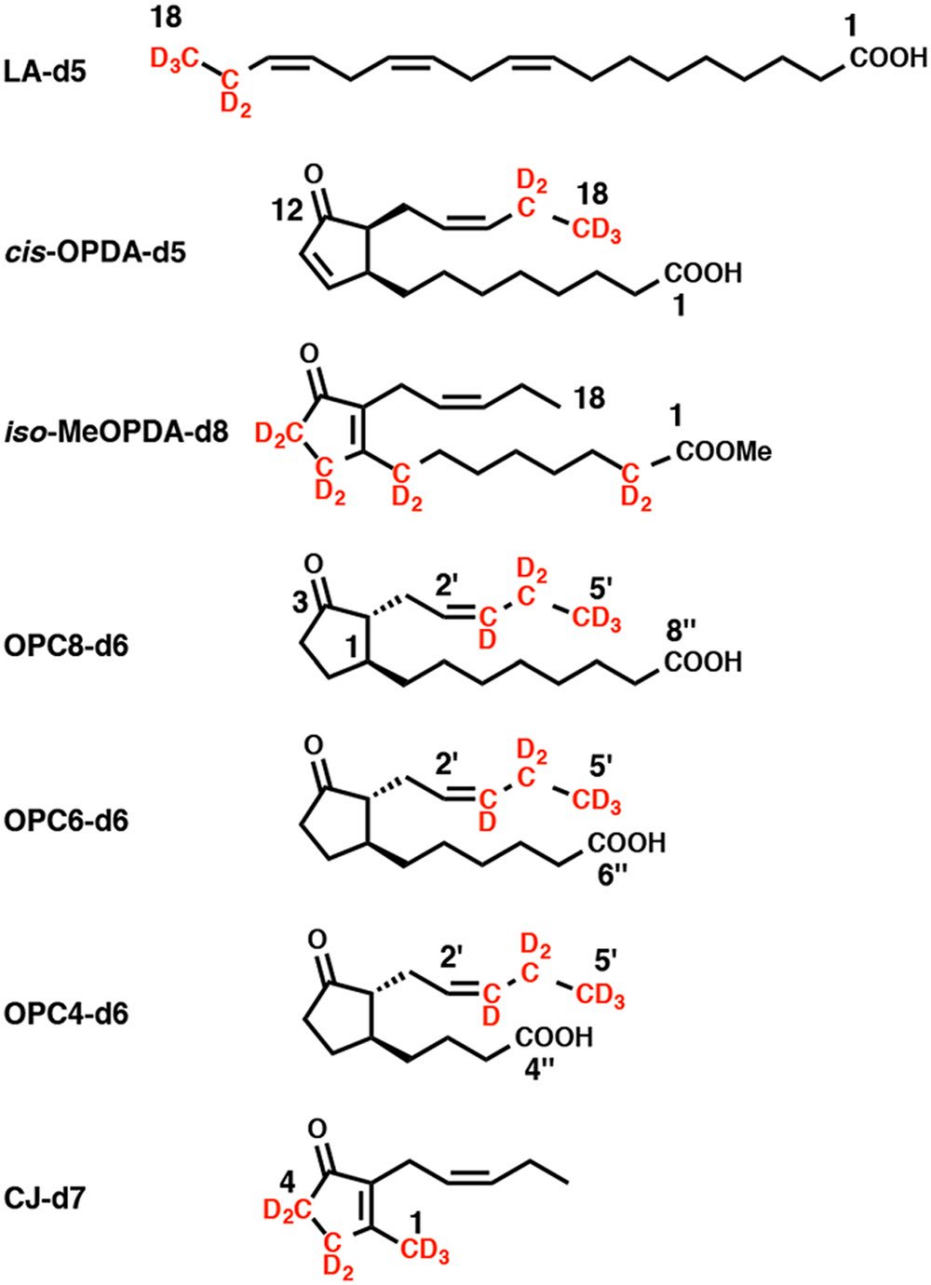
Chemical structures of deuterium-labeled compounds used in this study. Way of numbering the carbon atoms of OPDA-d5 was according to that of previous report^21^, and those of OPC8-d6, OPC6-d6, and OPC4-d6 were according to the report of Matsuura *et al*.^36^.

**Figure 4:**
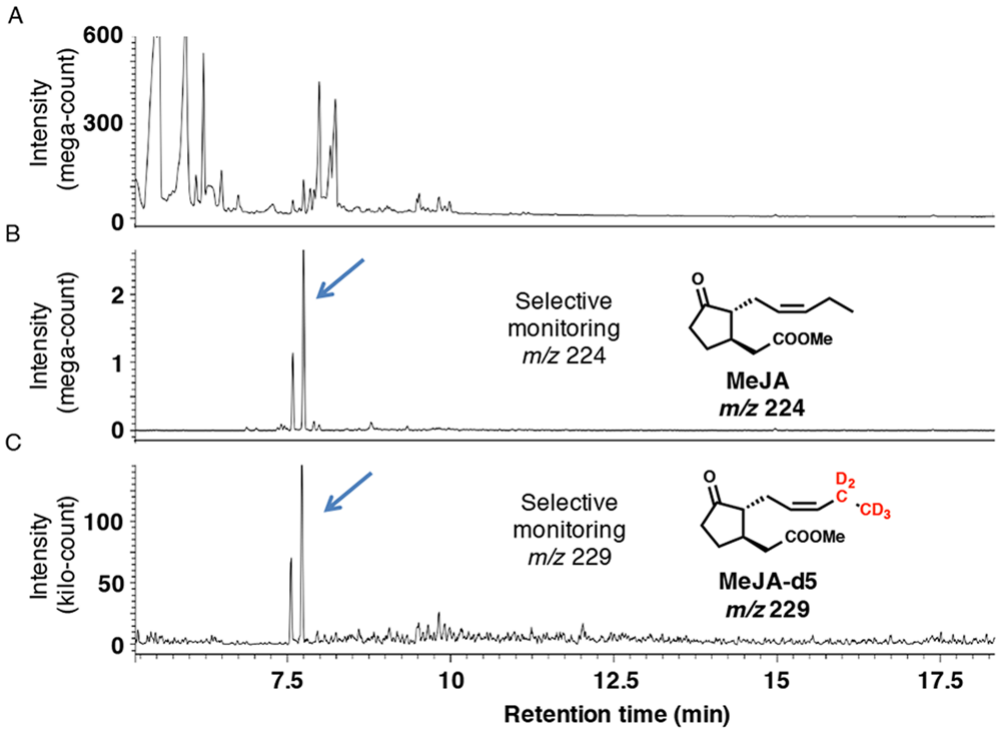
Representative GC-MS chromatograms for measuring MeJA in feeding experimet using LA-d5. (**A**) Representative GC-MS chromatogram monitoring total ion. (**B**) Representative GC-MS chromatogram for measuring fungal derived MeJA using selected ion monitoring at *m*/*z* 224. (**C**) Representative GC-MS chromatogram for measuring fungal derived MeJA-d5 using selected ion monitoring at *m*/*z* 229. Representative MS chart of the peak indicated by arrow is given in Supplementary Figure S4.

**Figure 5:**
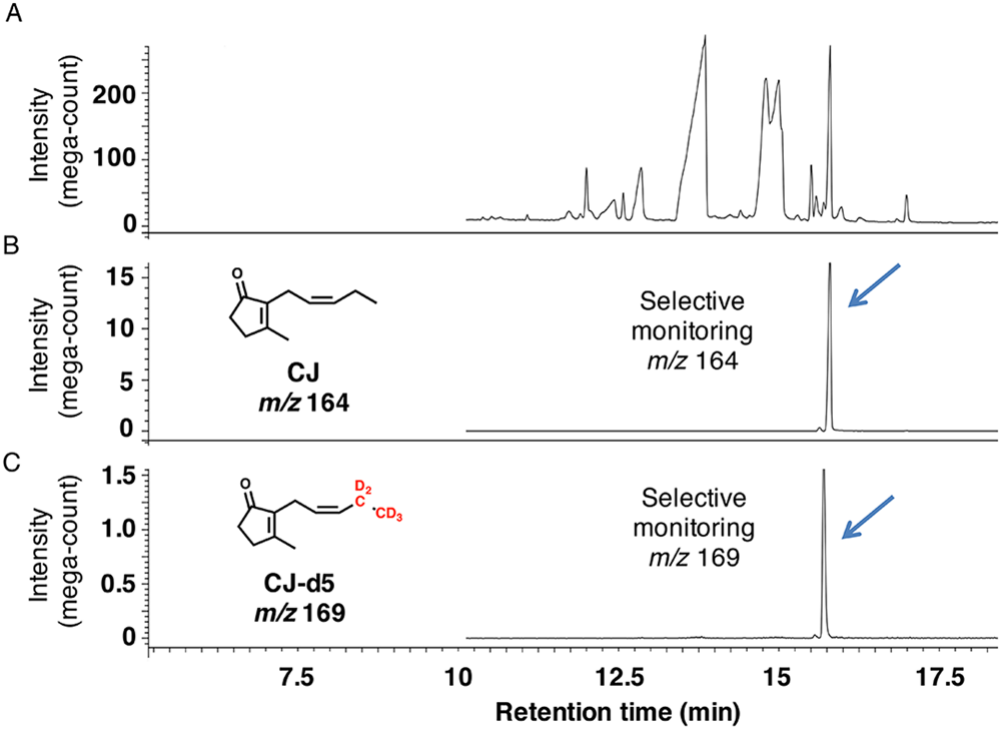
Representative GC-MS chromatograms for measuring CJ in feeding experiment using LA-d5. (**A**) Representative GC-MS chromatogram monitoring total ion. (**B**) Representative GC-MS chromatogram for measuring fungal derived CJ using selected ion monitoring at *m*/*z* 164. (**C**) Representative GC-MS chromatogram for measuring fungal derived CJ-d5 using selected ion monitoring at *m*/*z* 169. Representative MS chart of the peak indicated by arrow is given in Supplementary Figure S5.

CJ in plants is reportedly synthesized using JA and *iso*-OPDA as synthetic intermediates via independent pathways (pathways A and B, respectively, in Fig. 1) that utilize LA and *cis*-OPDA as common intermediates in an early biosynthetic step (Fig. 1)^13, 14^. Based on these reports, we hypothesized that *L*. *theobromae* also uses these pathways to produce CJ. To determine the biosynthetic pathway affording CJ, *L*. *theobromae* was administered deuterium-labeled intermediates, including [18,18,18-^2^H_3_, 17,17-^2^H_2_]LA (LA-d5), [18,18,18-^2^H_3_, 17,17-^2^H_2_]*cis*-OPDA (*cis*-OPDA-d5), [5′,5′,5′-^2^H_3_, 4′,4′-^2^H_2_, 3′-^2^H_1_]OPC 8:0 (OPC8-d6), [5′,5′,5′-^2^H_3_, 4′,4′-^2^H_2_, 3′-^2^H_1_]OPC 6:0 (OPC6-d6), [5′,5′,5′-^2^H_3_, 4′,4′-^2^H_2_, 3′-^2^H_1_]OPC 4:0 (OPC4-d6), and [11,11-^2^H_2_, 10,10-^2^H_2_, 8,8-^2^H_2_, 2,2-^2^H_2_]methyl *iso*-12-oxo-phytodienoate (*iso*-MeOPDA-d8) (Fig. 3). A schematic illustration of the experimental procedure is shown in Supplementary Fig. 2. *cis*-OPDA-d5 was synthesized using commercially available LA-d5 according to the method reported by Kajiwara *et al*.^35^, while OPC8-d6, OPC6-d6, and OPC4-d6 were synthesized according to Matsuura *et al*.^36^. CJ was detected in the extract without modification, although a portion of the EtOAc extract was treated with a CH_2_N_2_ solution to convert endogenous JA into MeJA to increase its volatility. Representative GC-MS chromatograms of authentic MeJA are shown in Supplementary Fig. 3. In the feeding experiment using LA-d5 as a substrate, the GC-MS chromatograms obtained in selected ion monitoring mode contained ion peaks at *m*/*z* 224 and 229 for MeJA and [12,12,12-^2^H_3_, 11,11-^2^H_2_]MeJA (MeJA-d5), respectively, at Rt. 7.5 min (Fig. 4). The mass fragmentation patterns for the *m*/*z* 224 and 229 ion peaks are shown in Supplementary Fig. 4 and substantiated the presence of MeJA and MeJA-d5 in the fungal culture filtrate. The GC-MS chromatograms for the analysis of CJ and [11,11,11-^2^H_3_, 10,10-^2^H_2_]CJ (CJ-d5) when using LA-d5 as a feeding substrate are shown in Fig. 5. These chromatograms contained ion peaks of *m*/*z* 164 and 169 for CJ and CJ-d5, respectively, which were detected at Rt. 15.7 min in selected ion monitoring mode. The mass fragmentation patterns of the *m*/*z* 164 and 169 ion peaks are shown in Supplementary Fig. 5 and confirmed the presence of CJ and CJ-d5 in the fungal culture filtrate. The results of the feeding experiment using *cis*-OPDA-d5 were identical to those of the LA-d5 experiment (data not shown).

### Deuterium-labeled OPC 8:0, OPC 6:0, and OPC 4:0 are metabolized to MeJA but not CJ

**Figure 6:**
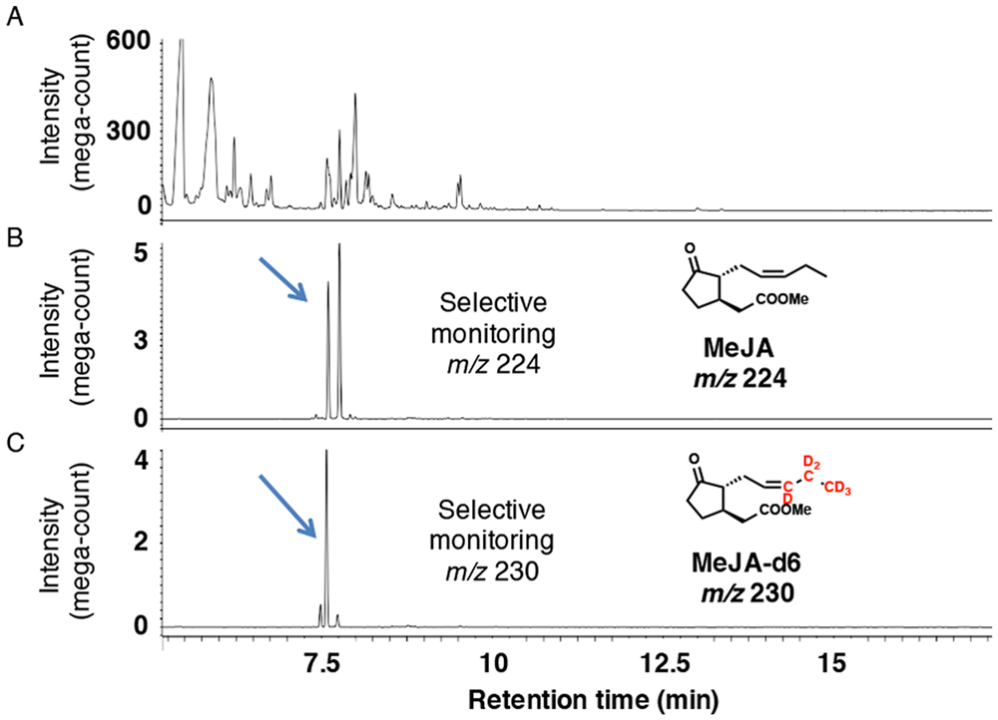
Representative GC-MS chromatograms for measuring MeJA in feeding experiment using OPC 8:0-d6. (**A**) Representative GC-MS chromatogram monitoring total ion. (**B**) Representative GC-MS chromatogram for measuring fungal derived MeJA using selected ion monitoring at *m*/*z* 224. (**C**) Representative GC-MS chromatogram for measuring fungal derived MeJA-d6 using selected ion monitoring at *m*/*z* 230. Representative MS chart of the peak indicated by arrow is given in Supplementary Figure S6.

**Figure 7:**
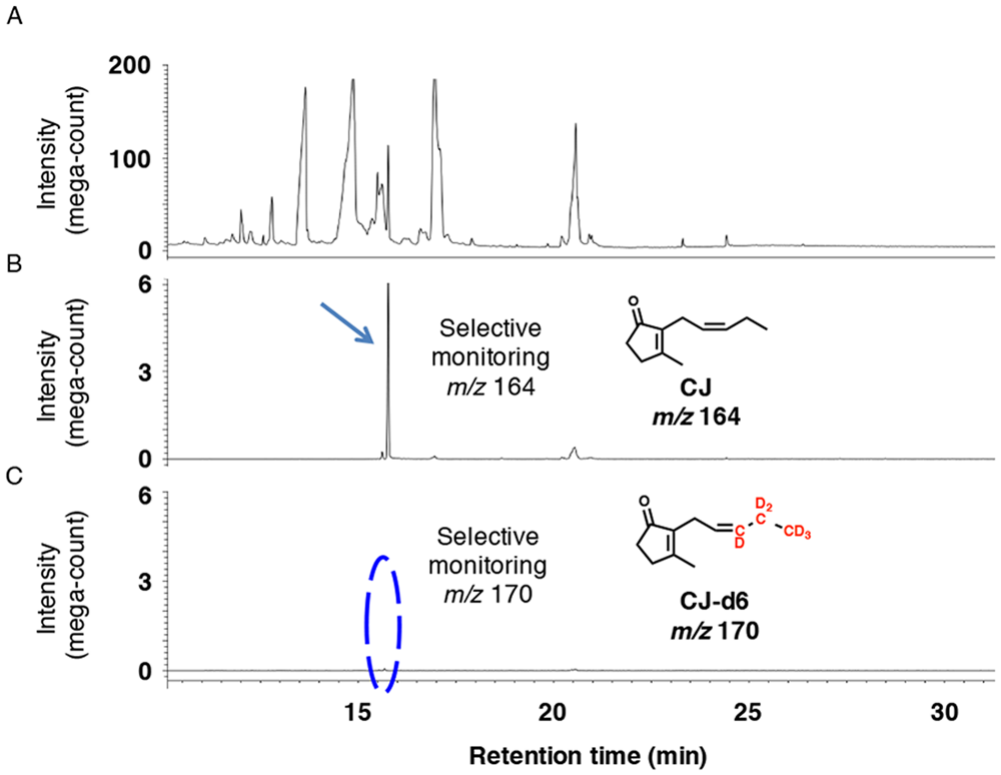
Representative GC-MS chromatograms for measuring CJ in feeding experiment using OPC8-d6. (**A**) Representative GC-MS chromatogram monitoring total ion. (**B**) Representative GC-MS chromatogram for measuring fungal derived CJ using selected ion monitoring at *m*/*z* 164. (**C**) Representative GC-MS chromatogram for measuring fungal derived CJ-d5 using selected ion monitoring at *m*/*z* 170. Representative MS chart of the peak indicated by arrow is given in Supplementary Figure S7.

In the feeding experiment using OPC8-d6 as a substrate, the GC-MS chromatograms obtained in selected ion monitoring mode contained ion peaks at *m*/*z* 224 and 230 for MeJA and [12,12,12-^2^H_3_, 11,11-^2^H_2_, 10-^2^H_1_]MeJA (MeJA-d6), respectively, at Rt. 7.5 min (Fig. 6). The mass fragmentation patterns for the *m*/*z* 224 and 230 ion peaks are shown in Supplementary Fig. 6 and confirmed the presence of MeJA and MeJA-d6 in the fungal culture filtrate. The GC-MS chromatograms for the analysis of CJ and [11,11,11-^2^H_3_, 10,10-^2^H_2_, 6-^2^H_1_]CJ (CJ-d6) when using OPC8-d6 as a feeding substrate are shown in Fig. 7. The *m*/*z* 164 ion peak was detected at Rt. 15.7 min in the selected ion monitoring chromatogram (Fig. 7B), although the identical ion peak was not detected in the chromatogram obtained from selected ion monitoring at *m*/*z* 170 (Fig. 7C). The mass fragmentation pattern of the ion peak at *m*/*z* 164 is shown in Supplementary Fig. 7 and confirmed the presence of CJ in the fungal culture filtrate. Because the *m*/*z* 170 ion peak could not be detected in a GC-MS chromatogram of acceptable quality (Fig. 7C), it was concluded that OPC8-d6 was not used as a building block to synthesize CJ-d6. The results of the feeding experiments using OPC6-d6 and OPC4-d6 were identical to those obtained using OPC8-d6 (data not shown). The results of the deuterium-labeled OPC 8:0, OPC 6:0, and OPC 4:0 feeding experiments thus suggested that biosynthetic pathway A was not involved in CJ production in *L*. *theobromae* (Fig. 1).

### Feeding deuterium-labeled *iso*-MeOPDA to growing fungal cultures produces labeled CJ as a metabolite but not labeled MeJA

**Figure 8:**
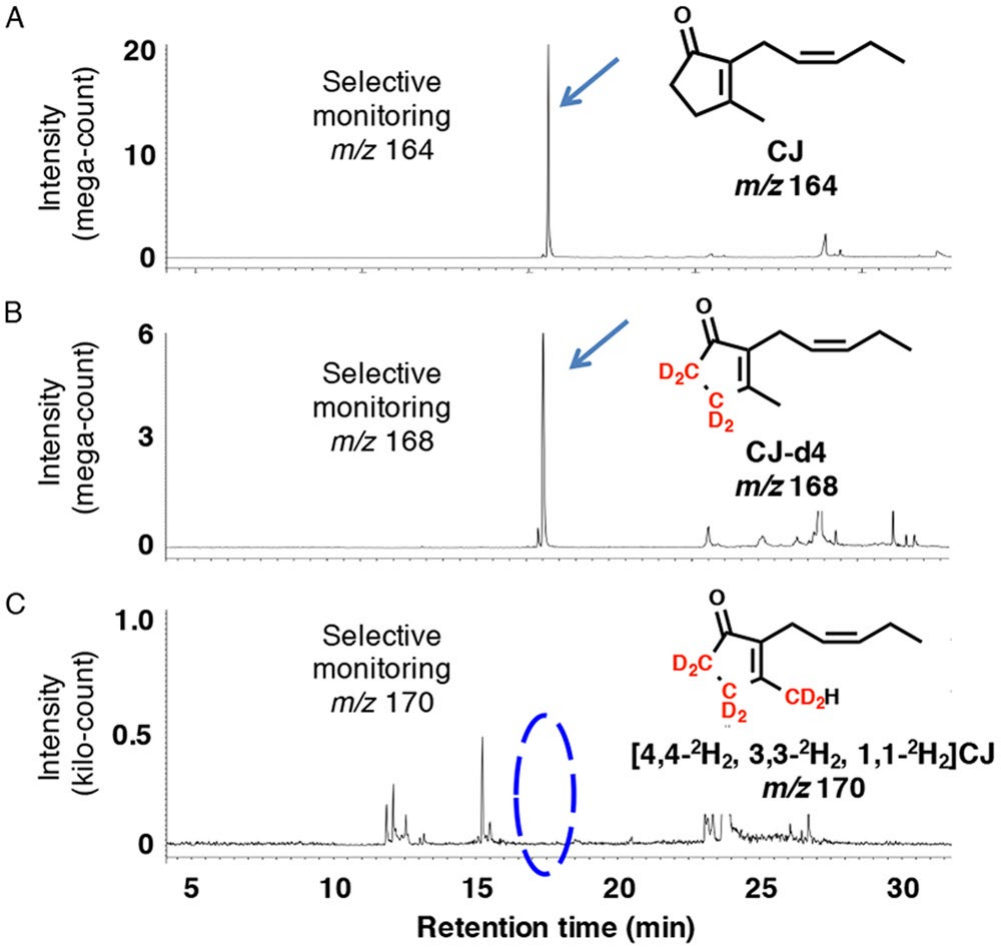
Representative GC MS/MS chromatograms analyzing fungal derived. CJ, CJ-d4, and CJ-d6 using *iso*-OPDA-d8 as a substrate for a feeding experiment. (**A**) Representative GC-MS chromatogram for measuring fungal derived CJ using selected ion monitoring at *m*/*z* 164. (**B**) Representative GC-MS chromatogram for measuring fungal derived CJ-d4 using selected ion monitoring at *m*/*z* 168. (**C**) Representative GC-MS chromatogram for measuring fungal derived CJ-d6 using selected ion monitoring at *m*/*z* 170. Representative MS chart of the peak indicated by arrow is given in Figure S8.

Plants also use *iso*-OPDA as a key intermediate in biosynthetic pathway B to synthesize CJ^14^. Furthermore, it has been proposed that the final step in establishing the chemical backbone of CJ is a decarboxylation reaction in which a carboxyl group is eliminated from 3,7-didehydroJA. In a previous report demonstrating biosynthetic pathway B in plants, [16-^2^H_1_, 15-^2^H_1_]*iso*-OPDA (*iso*-OPDA-d2) was synthesized and administered to the plants to demonstrate CJ metabolism. However, the final decarboxylation step was not fully proven. To examine biosynthetic pathway B in *L*. *theobromae* as well as the decarboxylation step, an experiment to administer [11,11-^2^H_2_, 10,10-^2^H_2_, 8,8-^2^H_2_, 2,2-^2^H_2_]methyl *iso*-12-oxo-phytodienoate (*iso*-MeOPDA-d8) was carried out. The carbon backbone of *iso*-MeOPDA-d8 was synthesized according to a reported method^37^, and deuterium labels were added to the product according to the reported method^34^ with some modifications, as described in the experimental section. The isotopic purity was established by comparing the data of GC-MS for *iso*-MeOPDA and *iso*-MeOPDA-d8. The incorporation of ^2^H_2_ was found to be 72% for *iso*-MeOPDA-d8. The synthesized compound was fed to *L*. *theobromae*, and the culture filtrate was analyzed by GC-MS. A representative GC-MS chromatogram is shown in Fig. 8. The *m*/*z* 164 and 168 ion peaks were detected at Rt. 15.7 min in selected ion monitoring mode (Fig. 8A and B, respectively). However, the predicted ion peak for *m*/*z* 170 was not detected (Fig. 8C). The mass fragmentation patterns of the *m*/*z* 164 and 168 ion peaks are shown in Supplementary Fig. 8 and confirmed the presence of CJ and [4,4-^2^H_2_, 3,3-^2^H_2_]CJ (CJ-d4) in the fungal culture filtrate. The labeled CJ-d4 pattern was determined and had specific characteristics. Ion peaks for a [M-CH_3_]^+^ fragment were observed in the CJ and CJ-d4 MS chromatograms (Supplementary Fig. 8), and a [M-CD_3_]^+^ fragment was detected in CJ-d7 (*m*/*z* 171, Supplementary Fig. 9), indicating that deletion of the methyl group at the C-1 position had occurred during MS fragmentation. This type of deletion was also observed as [M-CH_3_]^+^ from CJ and [9-^2^H_1_, 8-^2^H_1_]CJ and [M-CD_2_H]^+^ from [4,4-^2^H_2_, 1,1-^2^H_2_]CJ in the previous report^9^. These results supported the detection of CJ-d4 as [4,4-^2^H_2_, 3,3-^2^H_2_]CJ. Based on the abovementioned results, it appeared reasonable to suppose that CJ was synthesized via biosynthetic pathway B, and the predicted decarboxylation reaction might not be involved in the final biosynthetic step to afford CJ in *L*. *theobromae*. Interestingly, [5,5-^2^H_2_, 4,4-^2^H_2_, 2,2-^2^H_2_]MeJA (*m*/*z* 230) was not detected in the GC-MS data (Supplementary Fig. 10).

In this paper, it was found that *L*. *theobromae* produced CJ, although *B*. *cinerea*, *V*. *longisporum*, *F*. *oxysporum*, *G*. *fujikuroi*, and *C*. *heterostrophus* did not (Fig. 2). It appears that the ability to produce CJ is a unique feature of *L*. *theobromae*. In addition, it was suggested that the fungus used biosynthetic pathway B (Fig. 1) to produce CJ, and the predicted decarboxylation reaction might not be involved in the final biosynthesis step to produce fungal-derived CJ, suggesting the involvement of an unknown step. However, it has been generally accepted that the deuterium atoms at the α-position tend to be removed by enolization, and the α-methylene group at the C-2 position in 3,7-didehydroJA, which is strongly acidic due to the carboxyl groups, is a typical example of where this type of enolization can occur. However, Koch *et al*.^13^ have proven that deuterium-labeled atoms at the α-positions in [7-^2^H_1_, 5,5-^2^H_2_, 2,2-^2^H_2_] JA remain throughout the biosynthetic steps to give [4,4-^2^H_2_, 1,1-^2^H_2_]CJ in lima bean (*Phaseolus lunatus cv*. *Sieva*). Based on this report, it was presumed that the loss of deuterium atoms detected in the present study might not be due to enolization. To draw a more reliable conclusion, further research, such as feeding experiments applying [2-^13^C] and [1-^13^C, 2-^13^C]sodium acetate to fungal cultures of *L*. *theobromae*, is needed.

During our research, it was revealed that the fungus, *L*. *theobromae*, produced CJ. Since CJ has similar chemical structure with that of JA, it was assumed that CJ could mimic the biological activity of JA such as antagonistic effect upon salicylic acid signaling pathway^38^ that induces plant defense system. Given that CJ had JA like biological activity, *L*. *theobromae* might produce CJ to inhibit salicylic acid signaling pathway to facilitate invasion into the plants. However, this consideration was just hypothesis, and further research on the production of CJ in the fungus is needed to evaluate why the fungus synthesized CJ.

## Experimental Section

### General experimental procedures

NMR spectra were recorded in CDCl_3_ using a JNM-EX 270 FT-NMR spectrometer (JEOL) for ^1^H NMR experiments at 270 MHz and an AMX 500 (Bruker) for ^1^H NMR experiments at 500 MHz. FD-MS and FI-MS analyses were performed on a JMS-T100GCV (JEOL) instrument. GC-MS analyses were performed using a Varian instrument. Authentic CJ was purchased from Sigma-Aldrich. LA-d5 (98%) was purchased from Cambridge Isotope Laboratories, Inc.

### GC-MS conditions

GC-MS analysis of CJ and MeJA was completed on a Varian CP-3800 gas chromatograph with a Varian 1200 L quadrupole MS/MS in electron ionization mode. For the analysis of CJ, the injection temperature was 150 °C, and a fused-silica capillary column (TC-5; 30 m × 0.25 mm i.d., 0.25 μm film thickness; GL Sciences) was used. The temperature program started at  40 °C for 1 min and subsequently increased at 22 °C/min to 290 °C, which was maintained for 20 min. Helium was used as the carrier gas at a linear velocity of 1.2 mL/min, and all spectra were scanned within the range *m*/*z* 10–520. For the analysis of MeJA, the injection temperature was 250 °C, and a fused-silica capillary column (TC-5) was used. The temperature program started at  80 °C for 1 min and subsequently increased at 22 °C/min to 290 °C, which was maintained for 20 min.

### Identity of fungal sources

*B*. *cinerea* (MAFF No. 237695), *V*. *longisporum* (MAFF No. 243295), *G*. *fujikuroi* (MAFF No. 241712), *L*. *theobromae* (MAFF No. 306027), and *C*. *heterostrophus* (MAFF No. 243065) fungal cultures were obtained from The Genetic Resources Center, National Agriculture and Food Research Organization (NARO). The *F*. *oxysporum* culture (KF192-2) was a culture stock from the Laboratory of Plant Pathology, Research Faculty of Agriculture, Hokkaido University.

### Feeding experiments

The fungi were grown in 50 mL flasks containing 20 mL of 2% potato-sucrose stationary medium at 25 °C in the dark for 14 days. Deuterium-labeled compounds were dissolved in 1 mM aq. NH_4_OH (500 μL) and added to the medium at a final concentration of 1 mM. For extraction, 10 mL of EtOAc was added to the culture  together with addition of 1M aq. HCL (0.1 mL), and for the addition of internal standard, CJ-d7 (1 μg) was added to the separated EtOAc extract. The organic solvent in the extract were removed by a stream of N_2_. The resulting residues were dissolved in EtOAc. A portion of the EtOAc solution was subjected to GC-MS analysis to detect CJ, and another portion was treated with CH_2_N_2_ and subjected to GC-MS analysis to detect MeJA.

### [11,11-^2^H_2_, 10,10-^2^H_2_, 8,8-^2^H_2_, 2,2-^2^H_2_]methyl 12-oxophytodienoate (*iso*-MeOPDA-d8)

*iso*-MeOPDA (9 mg), which was synthesized according to a reported method^33^, was dissolved in CH_3_OD (1.0 mL) in an ampule; CH_3_ONa (4 mg) was then added to this solution. The ampule was sealed and heated at 65 °C for 5 days. The reaction mixture was quenched with 10% aq. NH_4_Cl and a small volume of water, and the solvent was removed under low pressure. Then, the residue was extracted with diethyl ether (10 mL × 3), and the solvent was removed under low pressure. The residue was purified by chromatography on silica gel (2 g) using ethyl acetate/*n*-hexane (1:4, v/v) to afford the product in a yield of 3.7 mg (35%). ^1^H NMR (500 MHz, CDCl_3_): δ 0.97 (t, J = 7.6 Hz, 3H), 1.31 (m, 6H), 1.49 (m, 2H), 1.58 (m, 2H), 2.13 (q, J = 7.4 Hz, 2H), 2.90 (d, J = 7.2 Hz, 2H), 3.64 (s, 3H), 5.19 (m, 1H), and 5.34 (m, 1H). FD-MS (*m/z*); 314 ([M]^+^ (100), 313 (31), 312 (6.3), 311 (2.1), which revealed the labeling ration of *iso*-MeOPDA-d8 was to be 72%.

### [4,4-^2^H_2_, 3,3-^2^H_2_, 1,1,1-^2^H_3_]CJ (CJ-d7)

A solution of CJ (51.6 mg) in CH_3_OD (1 mL) was added dropwise to a solution of CH_3_ONa (40 mg) in 2 mL of CH_3_OD. The mixture was refluxed for 5 days. NH_4_Cl was added, and the organic solvent was evaporated. The residue was dissolved in water (1 mL). After the product was extracted with Et_2_O (50 mL × 3), the organic layer was dried over Mg_2_SO_4_ and concentrated *in vacuo* after filtration. The residue was purified by flash column chromatography with silica gel (40 g) using ethyl acetate/*n*-hexane (1:9, v/v) to give [4,4-^2^H_2_, 3,3-^2^H_2_, 1,1,1-^2^H_3_]CJ in a yield of 53.7 mg (99%). GC-MS (m/z); 171 [M]^+^ (100), 170 (4.3), 169 (1.7), which revealed the labeling ration of CJ-d7 was to be 94%. ^1^H NMR (270 MHz, CDCl_3_): δ 0.96 (t, J = 7.0 Hz, 3H), 2.13 (m, 2H), 2.91 (d, J = 7.1 Hz, 2H), 5.24 (m, 1H), and 5.34 (m, 1H). FI-MS (*m*/*z*); [M^+^] 171.

## Electronic supplementary material

Supplementary Information
