# Supplementary material for: Elucidation of the biosynthetic pathway of cis-jasmone in Lasiodiplodia theobromae
Source: Sci Rep. 2017 Jul 27;7:6688. doi: 10.1038/s41598-017-05851-7 (PMC5532252; doi:10.1038/s41598-017-05851-7)
Supplement: Supplementary file 1 — Supplementary Information [file 41598_2017_5851_MOESM1_ESM.pdf]

Supplementary Information

Elucidation of the biosynthetic pathway of *cis*-jasmone in *Lasiodiplodia theobromae*

Ryo Matsui,[†] Naruki Amano,[†] Kosaku Takahashi,[†] Youdai Taguchi,[†] Wataru Saburi,[†] Hideharu Mori,[†] Norio Kondo,[†] Kazuhiko Matsuda[‡] and Hideyuki Matsuura*,[†]

[†]Research Faculty of Agriculture, Hokkaido University, Sapporo 060-8589, Japan
[‡]Graduate School of Agriculture, Faculty of Agriculture, Kinki University, Nakamachi, Nara 631-8505, Japan

List of Supplementary Information

Figure S1. Representative GC-MS chromatograms for measuring authentic CJ.

Figure S2. Experimental procedure for the feeding experiment.

Figure S3. Representative GC-MS chromatograms for measuring authentic MeJA.

Figure S4. Representative GC-MS chromatograms analyzing fungal-derived JA and JA-d5 using LA-d5 as a substrate for the feeding experiment.

Figure S5. Representative GC-MS chromatograms analyzing fungal-derived JA and JA-d5 using LA-d5 as a substrate for the feeding experiment.

Figure S6. Representative GC-MS chromatograms analyzing fungal-derived JA and JA-d6 using OPC8-d6 as a substrate for the feeding experiment.

Figure S7. Representative GC-MS chromatograms analyzing fungal-derived CJ and CJ-d6 using OPC8-d6 as a substrate for the feeding experiment.

Figure S8. Representative GC-MS chromatograms analyzing fungal-derived CJ and CJ-d4 using *iso*-OPDA-d8 as a substrate for the feeding experiment.

Figure S9. Representative GC-MS chromatogram analyzing authentic CJ-d7.

Figure S10. Representative GC-MS chromatographs for measuring MeJA in feeding experiment using *iso*-MeOPDA-d8.

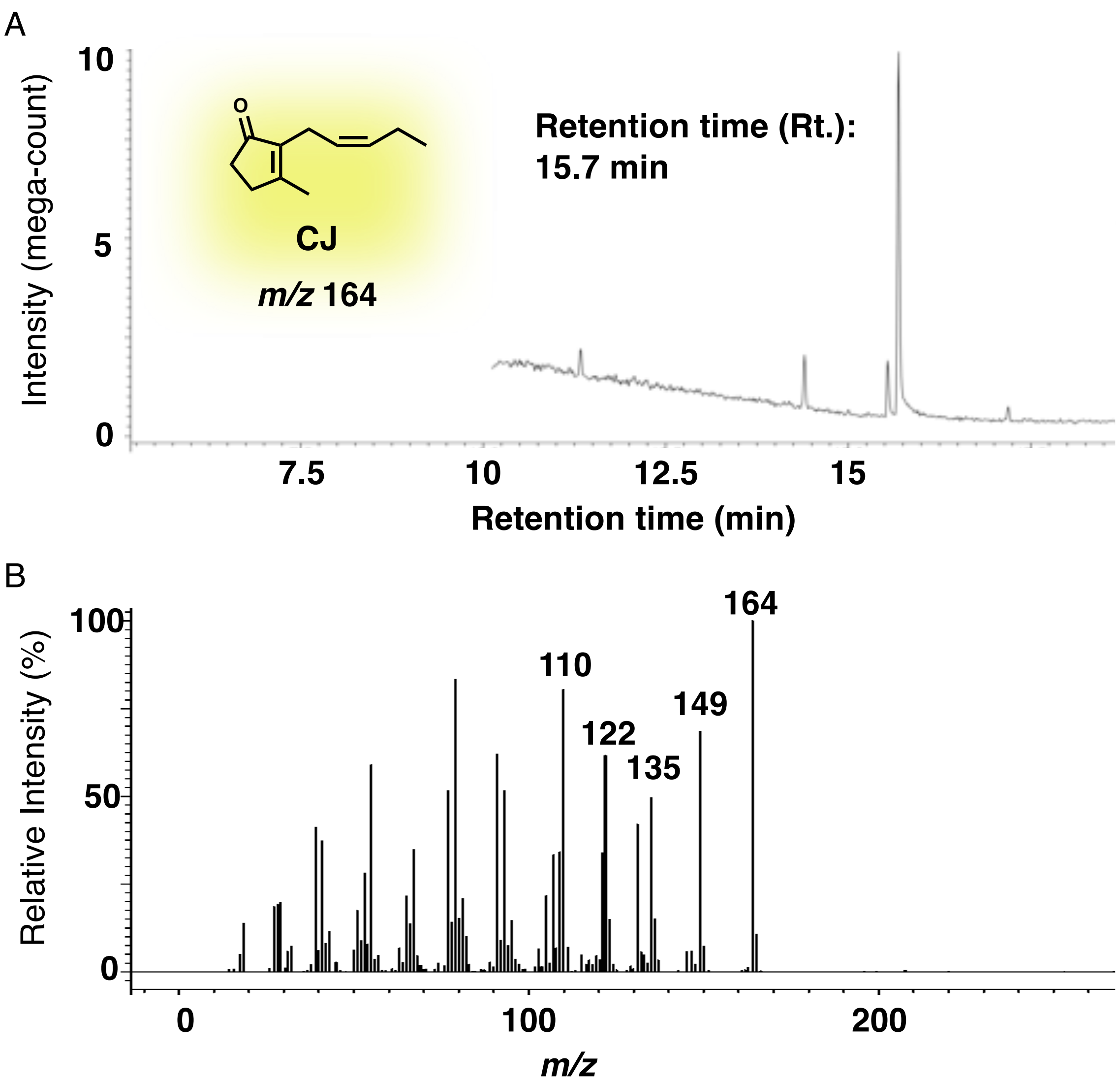

Figure S1. Representative GC-MS chromatograms for measuring authentic CJ.

A: Representative GC-MS chromatogram for measuring authentic CJ using selected ion monitoring at *m/z* 164. B: Fragmentation pattern of the MS peak having Rt. of 15.7 min in the chromatogram described in A.

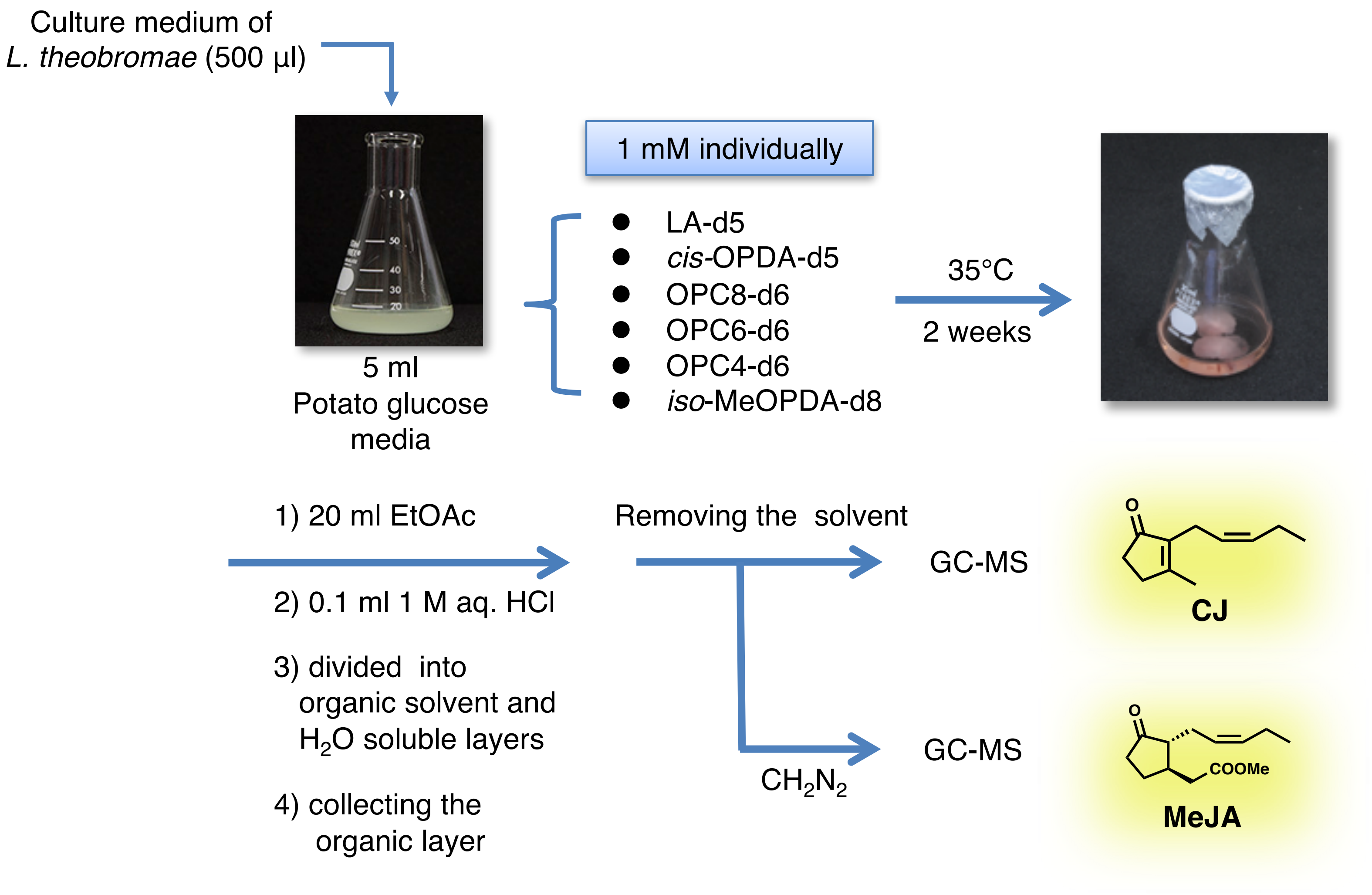

Figure S2. Experimental procedure for the feeding experiment.

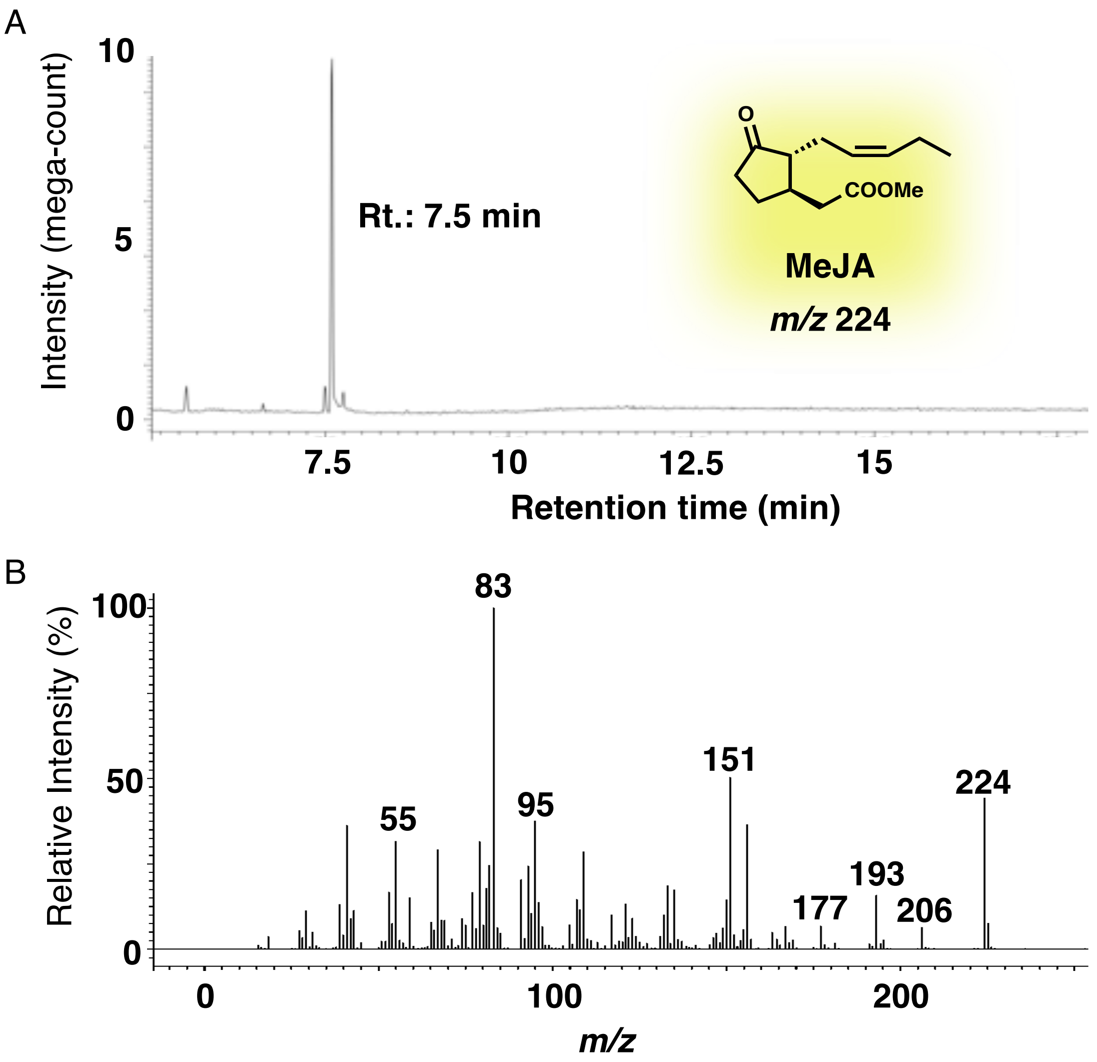

Figure S3. Representative GC-MS chromatograms for measuring authentic MeJA.
A: Representative GC-MS chromatogram for measuring authentic MeJA using selected ion monitoring at *m/z* 224. B: Fragmentation pattern of the MS peak having Rt. of 7.5 min in the chromatogram described in A.

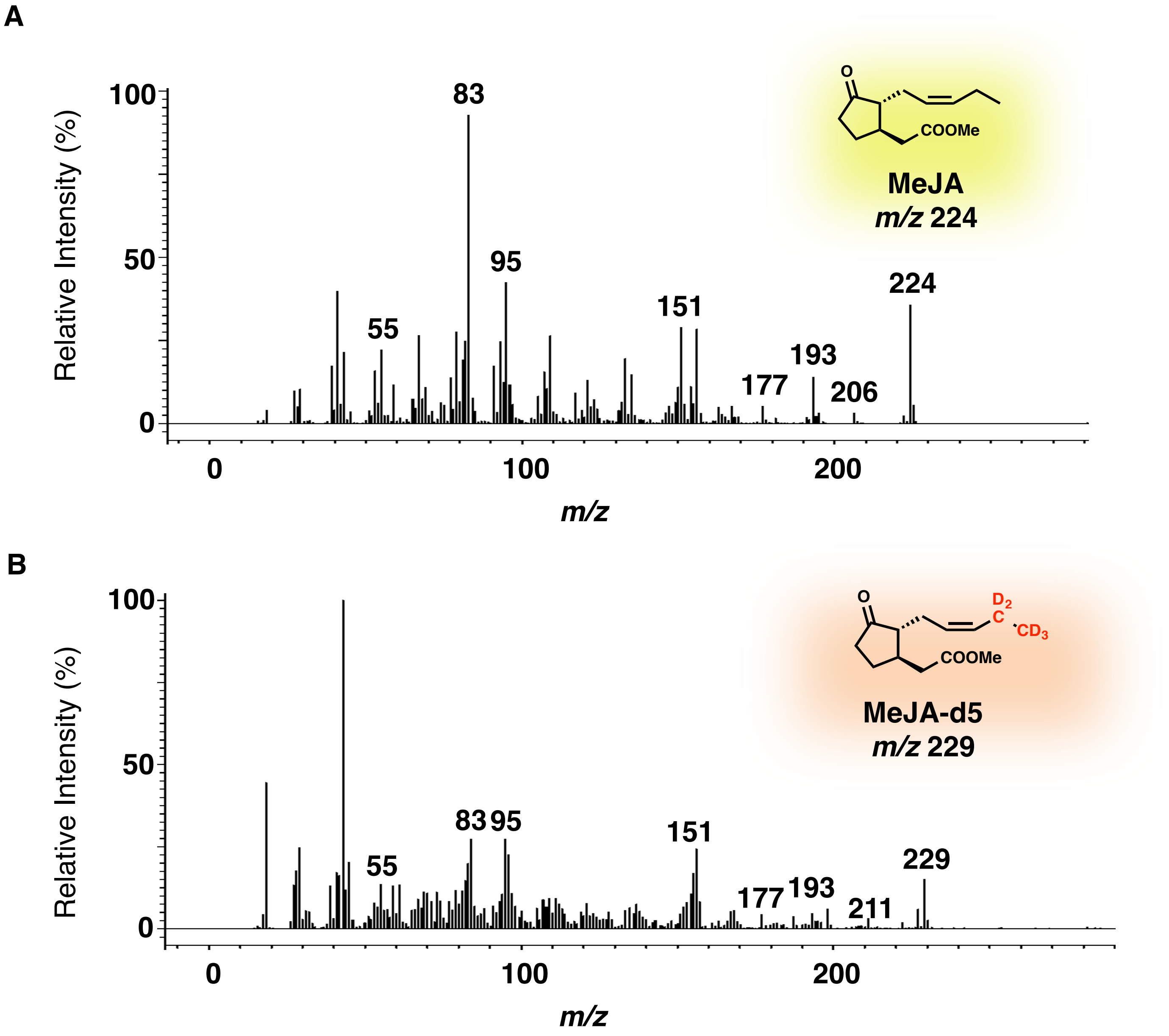

Figure S4. Representative GC-MS chromatograms analyzing fungal-derived JA and JA-d5 using LA-d5 as a substrate for the feeding experiment. A: MS chromatogram for analyzing MeJA. B: MS chromatogram for analyzing MeJA-d5.

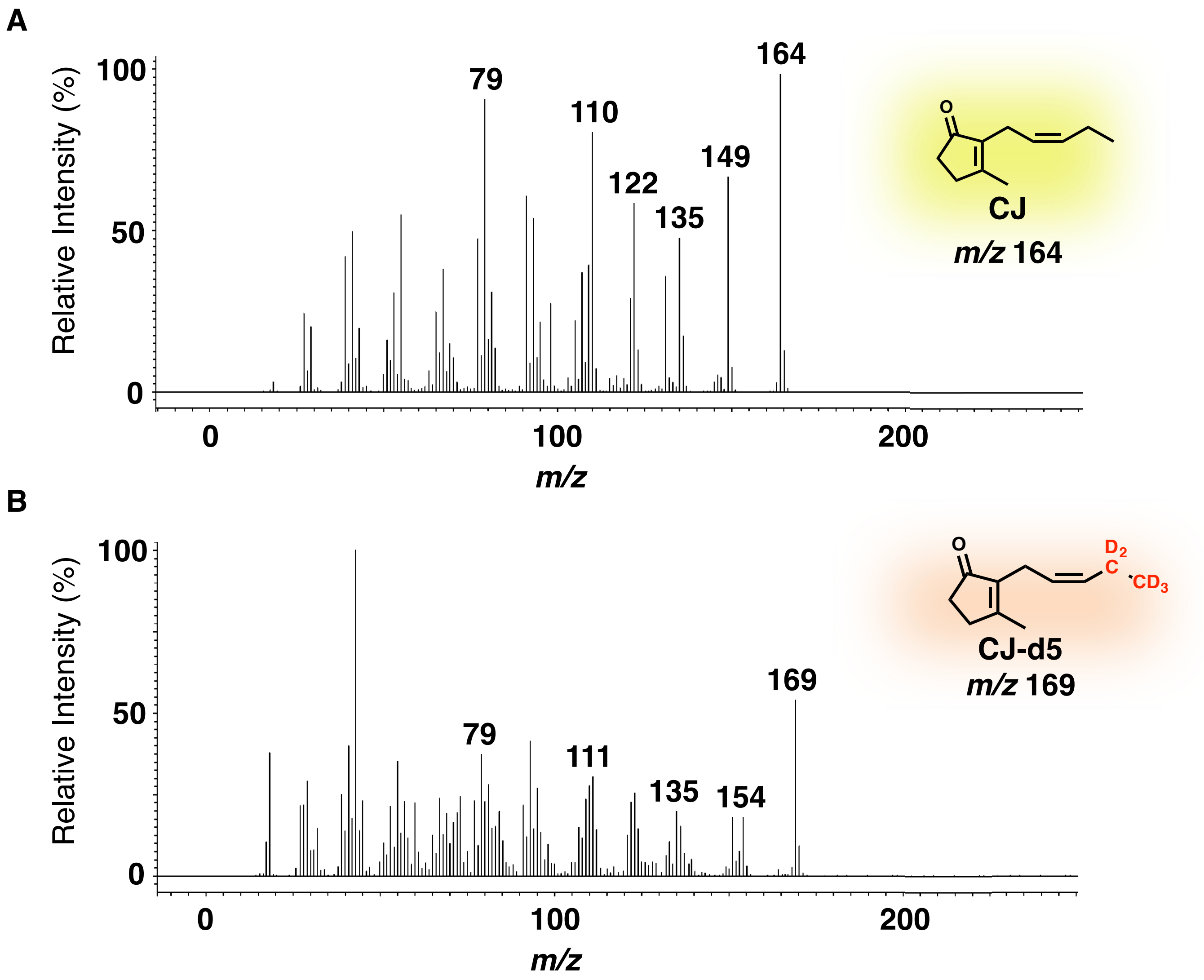

Figure S5. Representative GC-MS chromatograms analyzing fungal-derived JA and JA-d5 using LA-d5 as a substrate for the feeding experiment. A: MS chromatograph for analyzing CJ; B: MS chromatogram for analyzing CJ-d5.

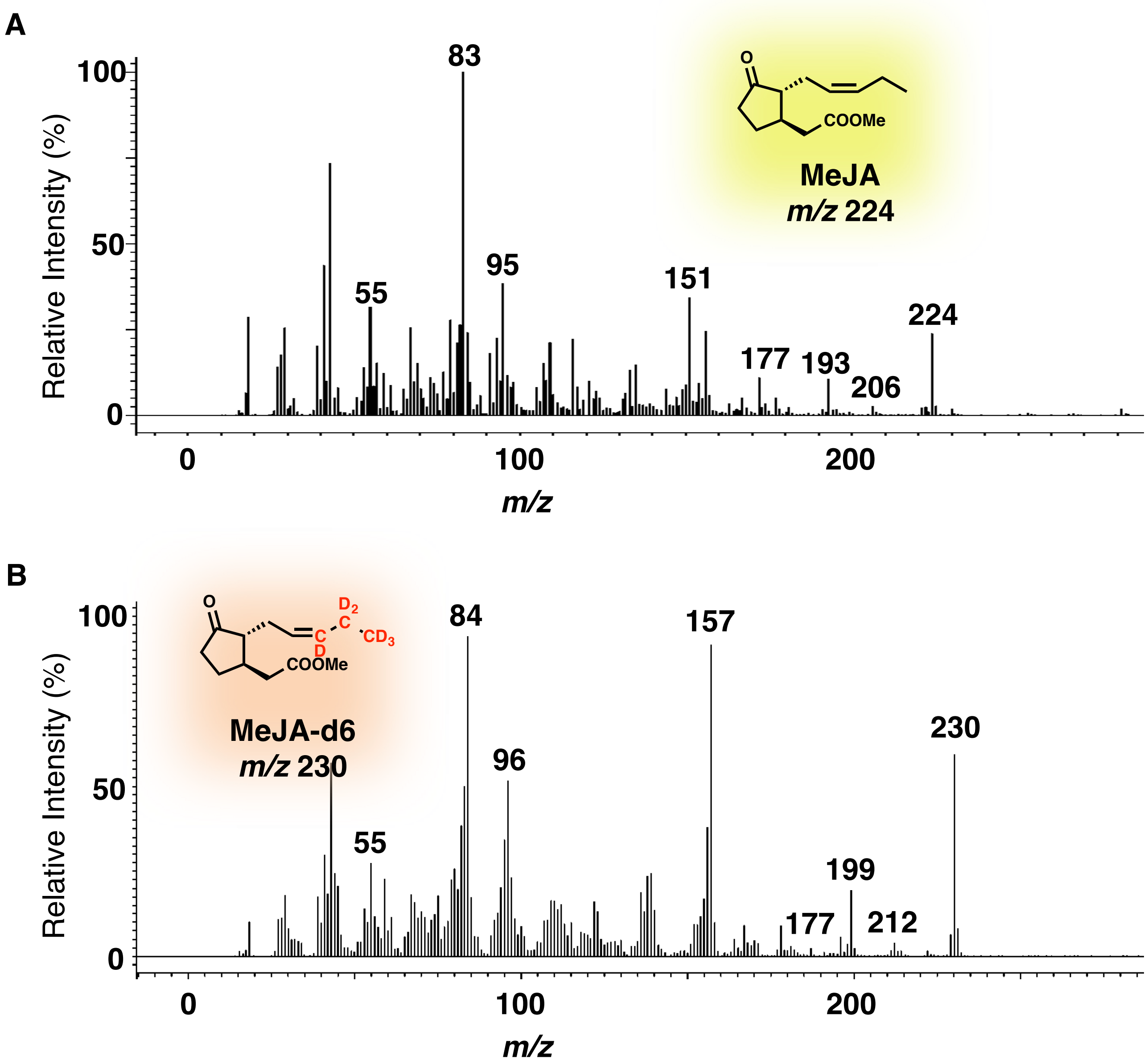

Figure S6. Representative GC-MS chromatograms analyzing fungal-derived JA and JA-d6 using OPC8-d6 as a substrate for the feeding experiment.

A: MS chromatogram for analyzing MeJA; B: MS chromatogram for analyzing MeJA-d6.

A

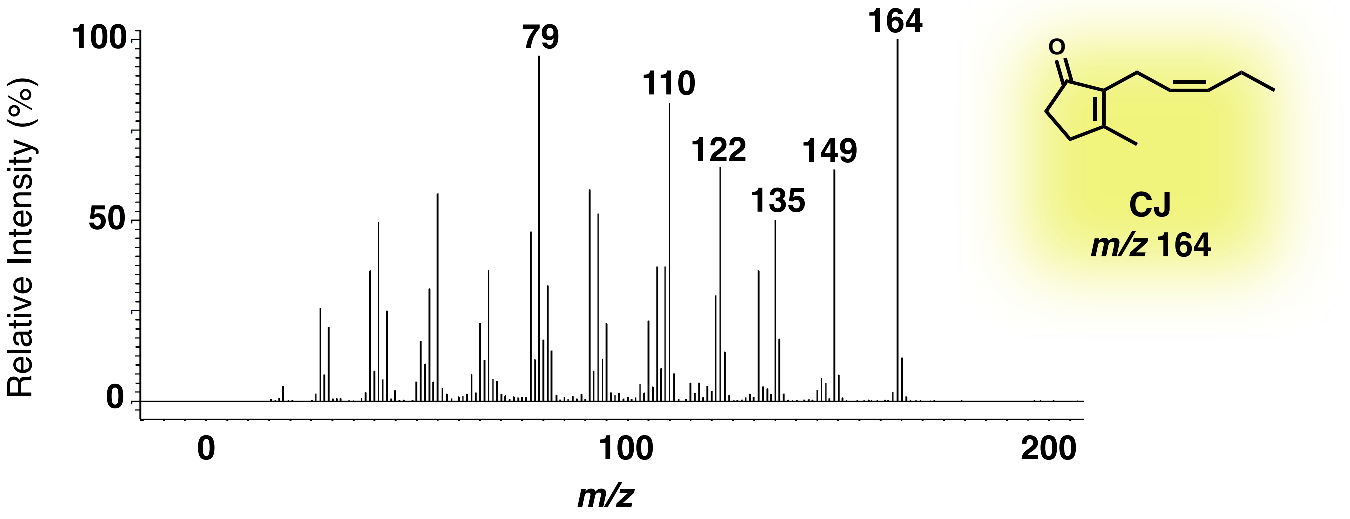

Figure S7. Representative GC-MS chromatogram analyzing fungal-derived CJ and CJ-d6 using OPC8-d6 as a substrate for the feeding experiment.
A: MS chromatogram for analyzing CJ.

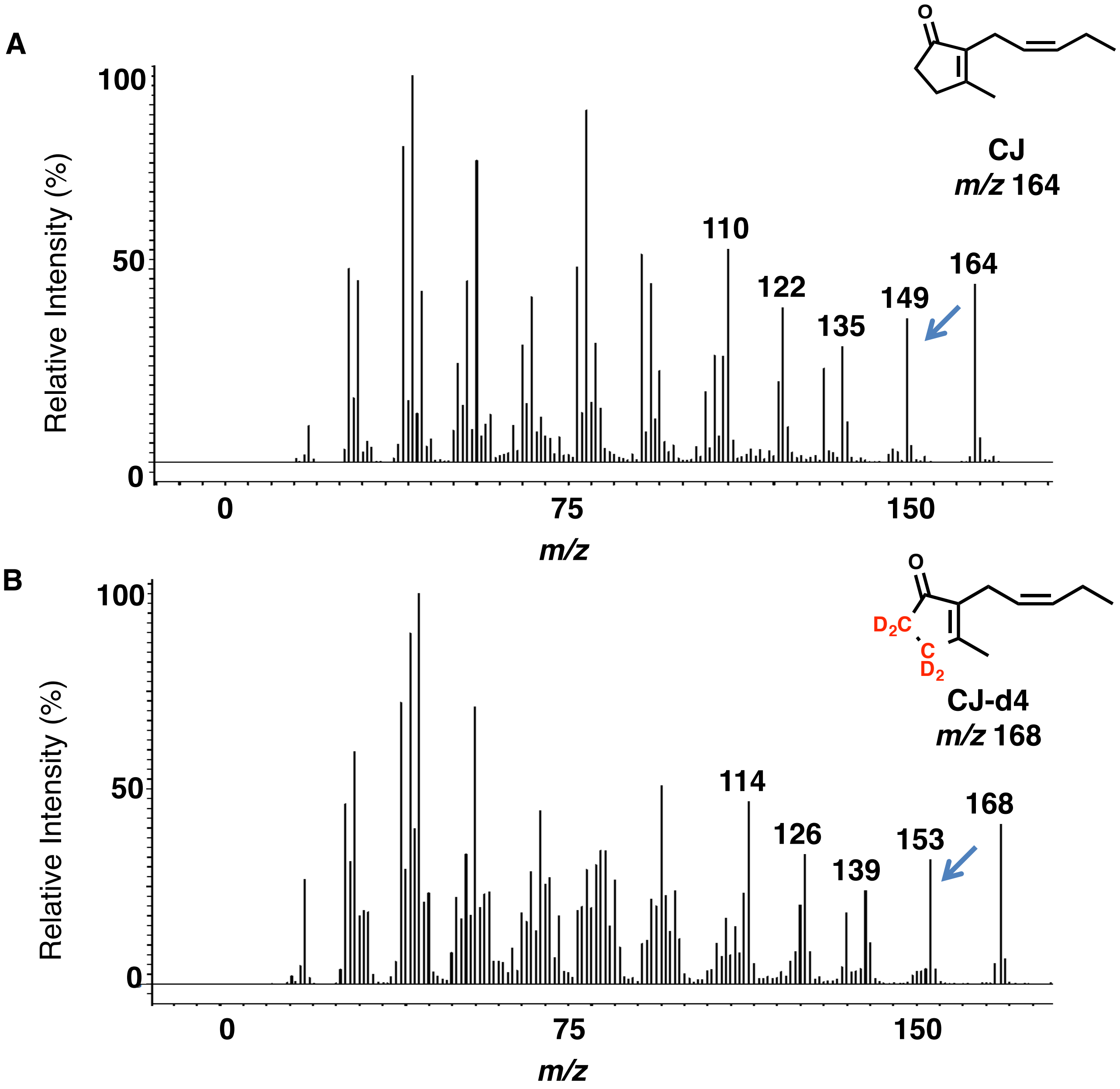

Figure S8. Representative GC-MS chromatograms analyzing fungal-derived CJ and CJ-d4 using *iso*-OPDA-d8 as a substrate for the feeding experiment.
A: MS chromatogram for analyzing CJ; B: MS chromatogram for analyzing CJ-d4.

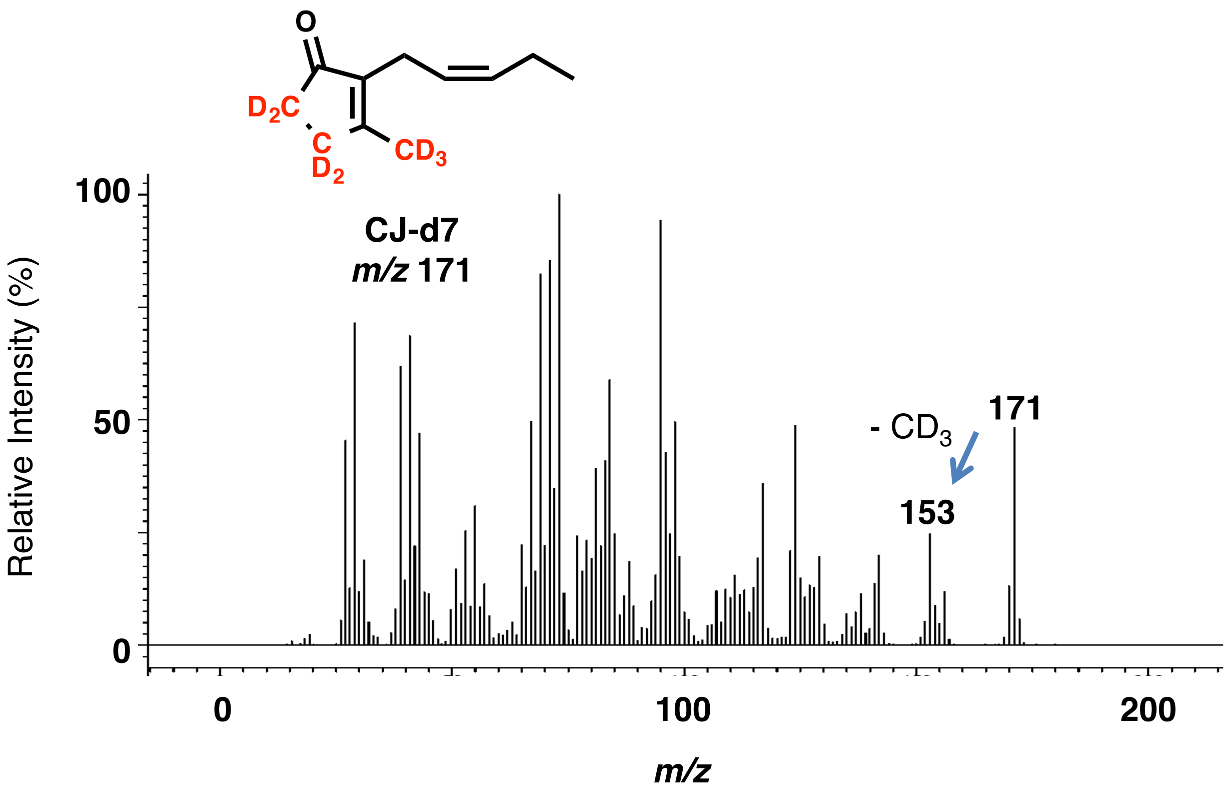

Figure S9. Representative GC-MS chromatogram analyzing authentic CJ-d7.

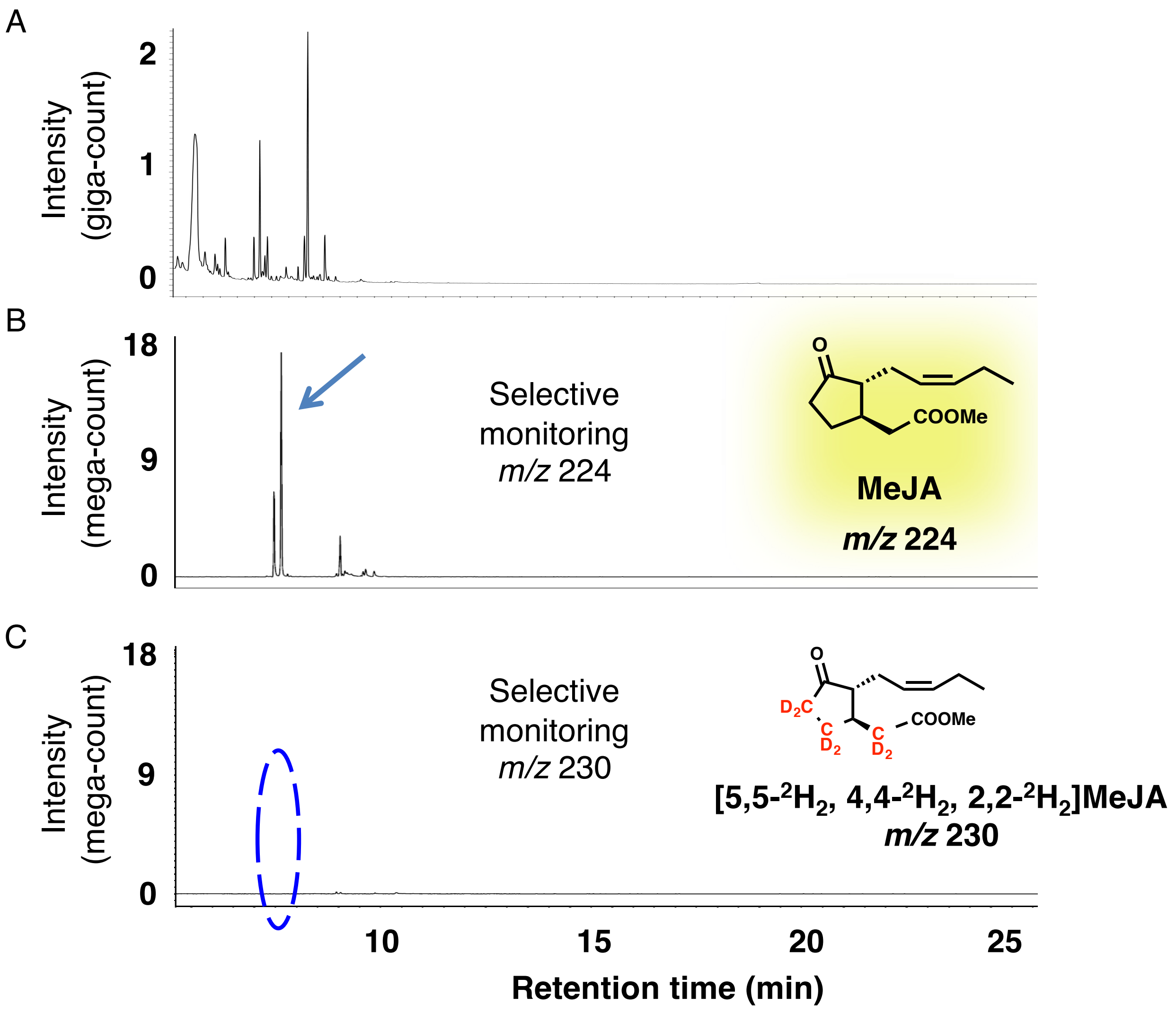

Figure S10. Representative GC-MS chromatograms for measuring MeJA in feeding experiment using *iso*-MeOPDA-d8. A: Representative GC-MS chromatogram monitoring total ion. B: Representative GC-MS chromatogram for measuring fungal derived MeJA using selected ion monitoring at *m/z* 224. C: Representative GC-MS chromatogram for measuring fungal derived [5,5-$^2H_2$, 4,4-$^2H_2$, 2,2-$^2H_2$]MeJA selected ion monitoring at *m/z* 230. MS chart of the peak indicated by arrow gives almost same feature with that of authentic MeJA given in Supplementary Figure S3.
